# Chemical synthesis of a two-photon-activatable chemokine and photon-guided lymphocyte migration *in vivo*

**DOI:** 10.1038/ncomms8220

**Published:** 2015-05-26

**Authors:** Xin Chen, Shan Tang, Ji-Shen Zheng, Ruozhu Zhao, Zhi-Peng Wang, Wen Shao, Hao-Nan Chang, Jing-Yuan Cheng, Hui Zhao, Lei Liu, Hai Qi

**Affiliations:** 1 Tsinghua-Peking Center for Life Sciences, Laboratory of Dynamic Immunobiology, Institute for Immunology, Tsinghua University, Beijing 100084, China; 2 Tsinghua-Peking Center for Life Sciences, Department of Chemistry, Tsinghua University, Beijing 100084, China; 3 High Magnetic Field Laboratory, Chinese Academy of Sciences, Hefei 230031, China; 4 Laboratory for Stem Cells and Epigenetics, School of Medicine, Tsinghua University, Beijing 100084, China; 5 School of Life Sciences, Tsinghua University, Beijing 100084, China

## Abstract

Chemokine-guided lymphocyte positioning in tissues is crucial for normal operation of the immune system. Direct, real-time manipulation and measurement of single-cell responses to chemokines is highly desired for investigating the cell biology of lymphocyte migration *in vivo*. Here we report the development of the first two-photon-activatable chemokine CCL5 through efficient one-pot total chemical synthesis in milligram scale. By spatiotemporally controlled photoactivation, we show at the single-cell level that T cells perceive the directional cue without relying on PI3K activities, which are nonetheless required for persistent migration over an extended period of time. By intravital imaging, we demonstrate artificial T-cell positioning in cutaneous tissues and lymph nodes. This work establishes a general strategy to develop high-quality photo-activatable protein agents through tailor-designed caging of multiple residues and highlights the potential of photo-activatable chemokines for understanding and potential therapeutic manipulation of cell positioning and position-controlled cell behaviours *in vivo*.

## Abstract (web summary)

The precise spatiotemporal control of chemokine exposure would be an advantageous tool for immune cell research. Here, Chen *et al.* develop a two-photon-activatable chemokine CCL5 and use it to direct lymphocyte migration *in vivo* and to show that PI3-kinase is not required to sense a gradient *in vitro*.

## Graphical abstract

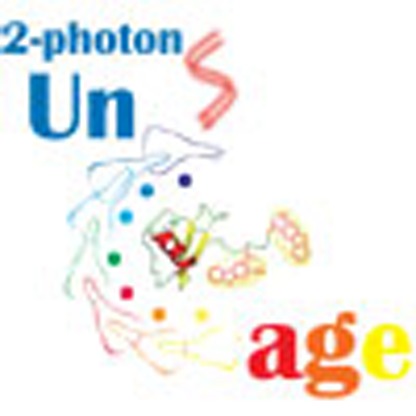

In serving host defense, lymphocytes must patrol potential sites of microbial invasion and rapidly locate locales of unpredictable tissue damage^1^. Chemokines play a key role in these processes, positioning lymphocytes under both quiescent and inflammatory conditions, allowing these migratory cells to interact with distinct microenvironments in spatiotemporally specific manners to develop and execute effector functions^2^. Our understanding of the chemosensing machinery is mainly derived from studies of *Dictyostelium* and neutrophils, and integrated models of intracellular signalling networks are being assembled to account for chemosensing sensitivity and robustness^3^. However, it is still being debated as to whether signal-centred models fully account for the range of biological behaviours observed with *Dictyostelium* and neutrophils^4^,^5^. It is even less clear whether lymphocyte behaviours obey the same paradigm, as different cell types may invoke different mechanisms to sense direction and orchestrate migration. Furthermore, chemokine-mediated recruitment of effector lymphocytes may be exploited to combat infections and tumours. Synthetic chemokines whose activities can be controlled in a spatiotemporally defined manner are a particularly attractive option to direct lymphocyte positioning and recruitment *in vivo*.

In this study, we have achieved total chemical synthesis and photocaging of human CCL5 (hCCL5) and use this model chemokine to establish a novel approach for studying lymphocyte chemosensing. Our results indicate phosphoinositide-3-kinase (PI3K) is not required for T cells to sense the directional cue *in vitro* and provide proof-of-concept demonstration for artificial control of lymphocyte localization *in vivo*.

## Results

### Total synthesis and validation of photoactivatable hCCL5

**Figure 1:**
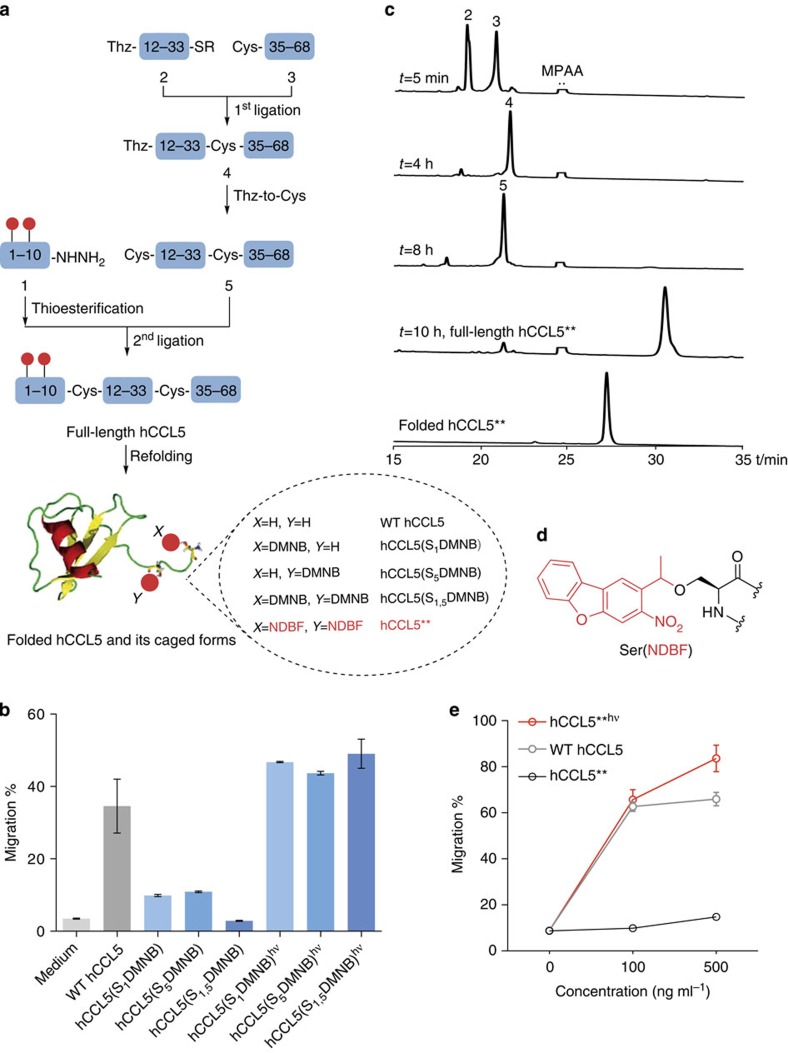
Total chemical synthesis and functional validation of wild-type and activity-caged hCCL5. (**a**) General strategy for chemical synthesis of hCCL5 and its photo-caged forms. (**b**) Transwell migration assay for wild-type hCCL5 and DMNB-caged versions before and after irradiation with ultraviolet light (365 nm) for 5 min. (**c**) HPLC traces for one-pot synthesis of hCCL5**. (**d**) Chemical structure of NDBF-modified Ser residue. (**e**) Transwell migration assay for two-photon caged hCCL5** before and after exposure to ultraviolet light (365 nm) for 30 s. Data representative of three independent experiments are shown both in **b** and **e**. Error bars indicate s.e.m. The image in **a** is modified from Protein Data Bank file 1RTO.

Mature, bioactive hCCL5 has 68 residues and is chemoattractive towards T cells, basophils and eosinophils through chemokine receptors CCR1, CCR3, CCR4 and CCR5 (ref. 6). To establish a practical procedure for full-length hCCL5 synthesis, we used a one-pot strategy^7^ that is based on 9-fluorenylmethoxycarbonyl (Fmoc) solid-phase peptide synthesis and native chemical ligation using modified thioester equivalents^8^,^9^,^10^. As shown in Fig. 1a, synthetic hCCL5 was made from three consecutive segments (Supplementary Fig. 1), namely [Ser^1^-Cys^10^]-NHNH_2_ (1), [Thz^11^-Lys^33^]^α^thioester (2) and [Cys^34^-Ser^68^] (3). The ligation of (2) and (3) in neutral aqueous buffer went to completion in 4 h to generate [Thz^11^-Ser^68^] (4). Addition of 0.25 M methoxyamine·HCl and pH adjustment to 4.0 for additional 4 h resulted in quantitative conversion of (4) to [Cys^11^-Ser^68^] (5). After pH re-adjustment to 6.8, the same batch was added with a slight excess of [Ser^1^-Cys^10^]^α^thioester, which was prepared by transthioesterification with 4-mercaptophenyacetic acid of (1). After 2 h, full-length [Ser^1^-Ser^68^] was generated. The product was purified and folded to afford hCCL5 in an overall isolated yield of 22% (Supplementary Fig. 2; mass: obsd (mean±s.e.m.=7845.2±0.7) Da; cald 7844.9 Da, average isotopes). Circular dichroism spectroscopy of the synthetic hCCL5 (Supplementary Fig. 3) was consistent with that of recombinant hCCL5 (ref. 11).

To cage the hCCL5 activity, we chose to modify two serine residues (Ser1 and Ser5) in the amino terminus by 4,5-dimethoxy-2-nitrobenzyl (DMNB) chemistry. Previous mutagenesis studies indicated that these residues are important for receptor binding and activation^12^. Two singly caged analogues hCCL5(S_1_DMNB) (mass: obsd (mean±s.e.m.=8040.4±0.8) Da; cald 8039.9 Da, average isotopes) and hCCL5(S_5_DMNB) (mass: obsd (mean±s.e.m.=8040.3±0.3) Da; cald 8039.9 Da, average isotopes), and a doubly caged analogue hCCL5(S_1,5_DMNB) (mass: obsd (mean±s.e.m.=8235.1±0.5) Da; cald 8235.0 Da, average isotopes) were successfully obtained using the same synthetic route as described above (Fig. 1a and Supplementary Fig. 2). Circular dichroism spectra of all the caged proteins showed a negative minimum at 203 nm, revealing that attachment of photosensitive DMNB groups to the N-terminal serine(s) did not appreciably change the overall hCCL5 structure (Supplementary Fig. 3). We then tested bioactivity of these synthetic hCCL5 proteins in a transwell migration assay using murine CD8^+^ T lymphocytes, which migrated towards hCCL5 in a CCR5-dependent manner (Supplementary Fig. 4). Whereas our synthetic native hCCL5 (Fig. 1b) exhibits activities indistinguishable from that of recombinant hCCL5 (not shown), chemotactic activities of the two singly caged forms were reduced but not abrogated (Fig. 1b). On the other hand, the doubly caged hCCL5 protein exhibited no measurable chemotactic activity. On exposure to the ultraviolet light (365 nm, 18 mW cm^−2^) for 5 min, the photolytic release of DMNB was complete (Supplementary Fig. 5a) and full chemotactic activity was restored for all the three caged analogues (Fig. 1b). Therefore, DMNB caging of both Ser 1 and Ser 5 generates fully caged and rapidly photoactivatable hCCL5. It is noteworthy that backbone caging was previously attempted to create a photoactivatable chemokine, but its uncaging required ultraviolet illumination for at least 60 min^13^, a process much slower than chemokine-regulated cell behaviours by orders of magnitude. By caging the chemokine–receptor interaction interface, our study establishes a general strategy to control chemokine activities with a very high temporal resolution.

With a practical uncaging method in hand, we recognized that illumination by the ultraviolet light is cytotoxic and unable to penetrate deep biological tissues. Intravital two-photon microscopy, owing to reduced photo-toxicities and tissue scattering of its long-wavelength excitation light, is particularly suited for studying immune cell migration and positioning dynamics *in vivo*^14^. Therefore, we tried caging of hCCL5 with a two-photon-sensitive protective group, nitrodibenzofuran (NDBF). NDBF exhibits excellent two-photon photochemical properties (two-photon cross-section=0.6 GM at 720 nm, half-time for diffraction-limited two-photon photolysis =0.3 ms) and has been successfully used to control Ca^2+^ release from the sarcoplasmic recticulum of intact cardiac myocytes^15^. We prepared Fmoc-Ser(NDBF)-OH (Supplementary Fig. 6 and Supplementary Methods) and, by Fmoc solid-phase peptide synthesis, obtained [Ser^1^-Cys^10^(Ser_1,5_NDBF)]-NHNH_2_. By the same synthetic strategy established in Fig. 1a, doubly NDBF-caged hCCL5 (denoted as hCCL5**) was successfully synthesized in multi-milligram scale (13% isolated yield, Fig. 1c,d and Supplementary Fig. 7) (mass: obsd (mean±s.e.m.=8322.6±0.3) Da; cald 8323.0 Da, average isotopes). Analysis of photolytic kinetics by the 365-nm ultraviolet light revealed that hCCL5** uncaging was completed within 30 s, which was almost ten times faster than that with DMNB (Supplementary Fig. 5b). Transwell assay confirmed that hCCL5** had essentially no chemotactic activity towards CD8^+^ T cells before ultraviolet illumination, but fully recovered the activity on irradiation (365 nm, 18 mW cm^−2^) for 30 s (Fig. 1e).

**Figure 2:**
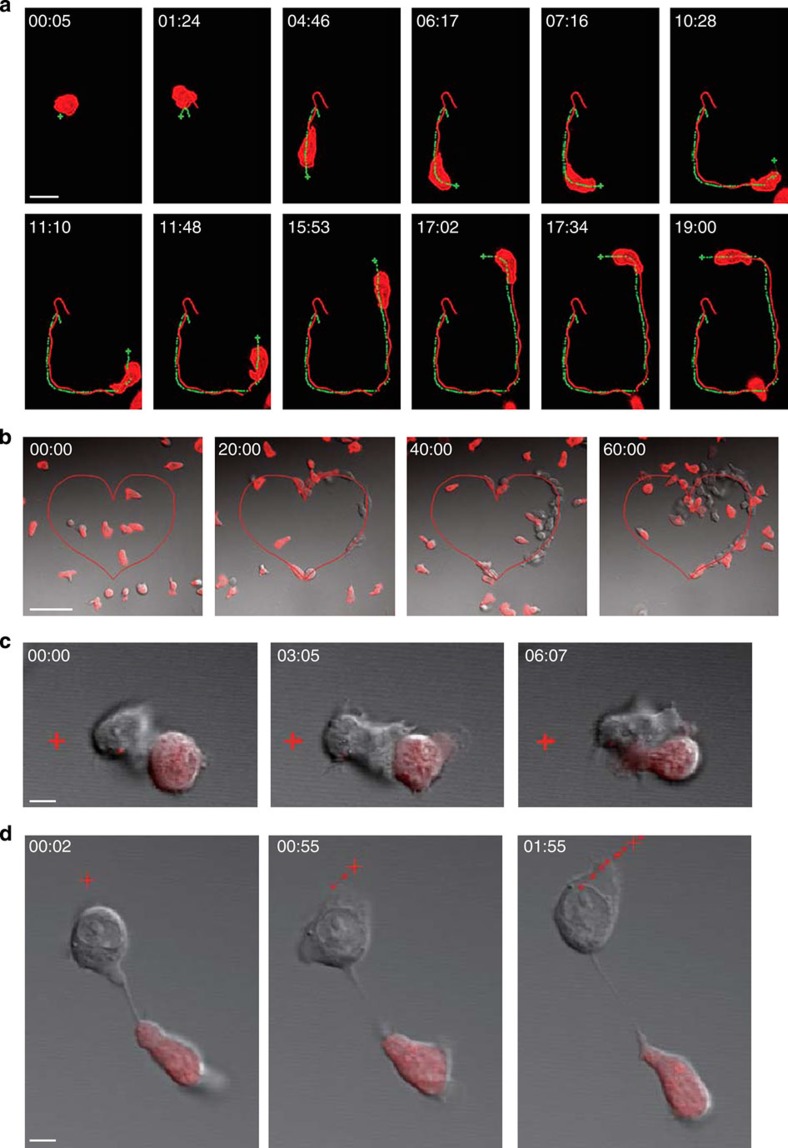
Two-photon guided T-cell migration *in vitro*. (**a**) Time-lapse images of a dsRed-expressing CD8^+^ T cell chasing the focus of a 720-nm two-photon excitation laser in media containing 1 μg ml^−1^ hCCL5**. The uncaging laser focus was manually placed in front of the leading edge. Green dots: previous positions of the laser focus; green cross: current focus at the indicated time point; red dots: centroid positions of the cell. Scale bar, 20 μm. (**b**) Time-lapse images showing T-cell accumulation over time towards the uncaging light path patterned in a heart shape (red line) in hCCL5**-containing media. Scale bar, 50 μm. Data representative of more than five independent experiments. Time-lapse images showing a stable (**c**) or unstable (**d**) contact engaged CD8^+^ T-cell response to hCCL5 attraction delivered by a focus of 720-nm two-photon excitation continuously. Red dots: previous positions of the laser focus; red cross: current focus at the indicated time point. Scale bar, 5 μm. Data are representative of more than five independent experiments.

Next, we tested two-photon uncaging of hCCL5** using video microscopy. As shown in Fig. 2a and Supplementary Movie 1, a CD8^+^ T cell placed in media containing hCCL5** on the ICAM-2-coated plate was guided by the focus of a 720-nm laser as it was manually moved across the field. Complex patterns of T-cell localization can be achieved with freely definable uncaging light paths, as exemplified by the heart-shaped configuration in Fig. 2b and Supplementary Movie 2. In addition, to test the possibility of using photoactivatable hCCL5** to study chemotaxis or related cell behaviours in context of cell–cell interactions, we photoactivated hCCL5** at the opposite side of T cells engaged with antigen-presenting cells (APC). We observed that T cells stably engaged with the APC were insensitive to chemokine attraction (Fig. 2c and Supplementary Movie 3), only regaining the sensitivity on APC disassociation (Fig. 2d and Supplementary Movie 4). The temporary insensitivity is consistent with cytoskeleton rearrangement organized by T-cell receptor signalling and recruitment of chemokine receptors, probably CCR5, to the immunological synapse (Supplementary Fig. 4 and ref. 3). Therefore, by taking advantage of the rapid two-photon uncaging kinetics and developing the first activity-caged, two-photon-activatable chemokine, we have created a new way to deliver site-specific chemokine stimulation and manipulate directional T-cell migration in real time. A key aspect of this technology is the high efficiency and flexibility of the chemical synthesis scheme, which provides convenient access to tailor-designed caged proteins bearing modifications at multiple sites. More importantly, the synthetic method described here can be used to develop other activity-caged chemokines and other small- to medium-sized proteins for mechanistic and therapeutic studies.

### PI3K is required for migration but not directional sensing

**Figure 3:**
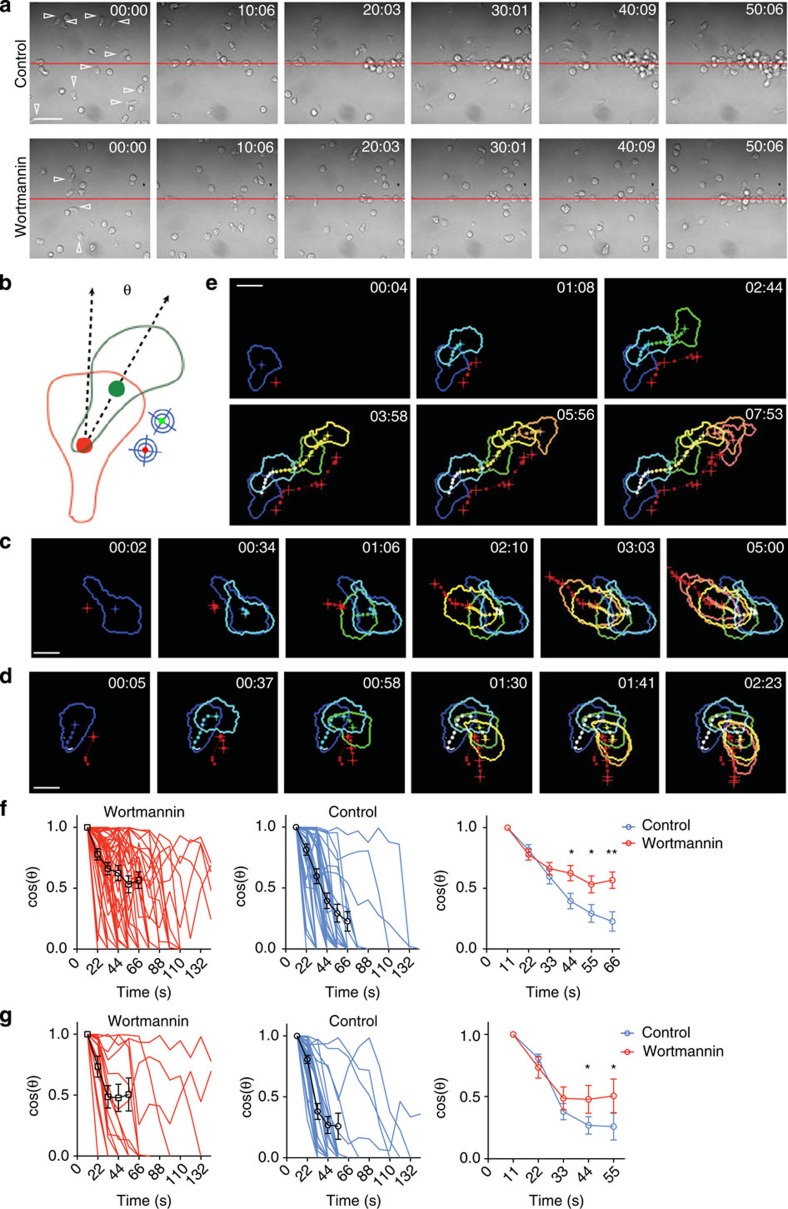
PI3K activities required for persistent migration but not directional sensing. (**a**) Time-lapse images showing directed migration and accumulation of T cells, untreated or treated with 100 nM wortmannin, in hCCL5**-containing media towards the uncaging light path patterned as a line. Open arrowheads: polarized cells with a shape index >1.5. Scale bar, 50 μm. (**b**) Schematic diagram of single T-cell chemotaxis assay by two-photon uncaging of hCCL5**. The focal point (∼0.078 μm^2^, crossed circles) always placed on one side of the cell. Angle *θ* is between the current instantaneous velocity vector and the velocity vector at the first time point after uncaging. The centroid positions of the cell are denoted with the red and green dots. Time-lapse images of T cells that, in response to active CCL5, generate new pseudopods (**c**, ‘N-turn') or extend existing pseudopods towards the uncaging light focus (**d**, ‘U-turn') or remain uncommitted for a prolonged period (**e**, ‘C-turn'); cell shapes outlined in colours of different warmth to indicate time and pseudopod dynamics. See corresponding Supplementary Movie 7–9. Scale bar, 10 μm. (**f**,**g**) Kinetic decay curves of cosine (*θ*). For each cell, values of cosine (*θ*) before it reaches zero were used in calculation. Cells whose cosine (*θ*) remained positive throughout the intial 200 s on uncaging were excluded. T cells untreated or treated with wortmannin (100 nM) at 10% (**f**) or 80% laser power output for uncaging (**g**); left and middle: curves for individual cells (coloured) and mean±s.e.m. (black); right: comparison of untreated and treated; *P<0.05, **P<0.01, by two-tailed *t*-tests.

A photo-activatable chemokine can be an excellent tool to study spatiotemporally controlled cell behaviours at the cellular level as well as signalling transduction at high spatiotemporal resolution at the molecular level. As a demonstration, we examined the role of PI3K in T-cell chemotaxis *in vitro*. Earlier studies of *Dictyostelium* and neutrophil migration indicate that PI3K is essential for sensing the chemokine gradient and driving directional migration^16^,^17^,^18^,^19^. More recent work, however, suggests that *Dictyostelium* cells or neutrophils deficient in PI3K activities are still capable of directional migration to certain extent, even though they exhibit reduced polarization, speed and fraction of responding cells^20^,^21^. Precisely, for what purpose PI3K is required during the process of eukaryotic chemotaxis is not fully understood^22^. Lymphocytes have rarely been studied in this regard, in part because of their small sizes and associated difficulties to manipulate, despite the fundamental importance of chemotaxis for their normal functions. A recent *in-vivo* study demonstrates that during the induction of a germinal centre response, migration of T lymphocytes from the T-cell zone into the B-cell follicle requires not only the chemokine receptor CXCR5 but also the inducible co-stimulator receptor that independently activates PI3K^23^, further raising the question as to how PI3K activities not associated with the guidance receptor *per se* contribute to the directional migration of T lymphocytes. We thus further explored the role of PI3K in T-cell chemotaxis *in vitro* using the newly established system. As shown in Fig. 3a (time 0), when T cells were briefly treated with 100 nM wortmannin, a majority of them became depolarized (the ratio between long and short axes of the cell, or shape index, <1.5), as can be expected^24^. However, these cells were still able to migrate, albeit with much reduced speeds, up to the CCL5 gradient and eventually accumulated at the source of active CCL5 to a significant degree (Fig. 3a and Supplementary Movie 5 and 6). These data suggest that similar to *Dictyostelium* cells or neutrophils^20^,^21^, T lymphocytes do not absolutely need PI3K activities for directional migration. However, at the population level it was difficult to determine whether this is because PI3K-inhibited T cells could not perceive the gradient as effectively or migrate as efficiently, or both. Therefore, to probe whether, beyond cellular polarization and maintaining migration speed, PI3K is required for additional processes involved in chemotaxis, we took advantage of the unique ability of our system to create a point source (<0.1 μm^2^) of bioactive chemokine, owing to the small volume of effective two-photon excitation and examined one-by-one behaviours of individual T cells that are polarized (shape index >1.5) despite PI3K inhibition. As shown in the diagram of Fig. 3b, we placed the point source by the side of the T cell roughly at the middle point between the front and the back and video-recorded cell migration to determine how fast a T cell could turn 90° and points its front towards the CCL5 point source. Three types of pseudopod behaviours were observed: some cells immediately generated a new front facing the point source (N-turn behaviour; Fig. 3c and Supplementary Movie 7) or extended its existing pseudopod front towards the point source and make a sharp turn (U-turn behaviour; Fig. 3d and Supplementary Movie 8), whereas additional cells exhibited significant ‘hesitation', alternatively extending pseudopods towards and away from the source for a significant period of time (C-turn behaviour; Fig. 3e and Supplementary Movie 9). Some T cells exhibited a hybrid response of mixed N- and C-turn (mix-turn behaviour; Supplementary Fig. 8). We calculated cos(θ) every 10 s for each cell and plotted it against time. The amount of time it takes for cos(θ) to decay from 1 to 0 is a universal measurement for how effectively the cell senses and executes the change of direction (Fig. 3b). As shown in Fig. 3f and Supplementary Fig. 8, wortmannin-treated cells changed directions as efficiently as the untreated cells in the first 30 s after chemokine exposure, but beyond this short period, even when the cell shape polarization was apparently normal, PI3K-inhibited cells were less efficient in following the point source of CCL5. These data suggest that in T lymphocytes, PI3K activities are not responsible for sensing the direction *per se* but for coordination of persistent movement to execute the migration towards the sensed direction. To test whether an increase in the strength of the CCL5 gradient could ameliorate the defect, we increased the power of the uncaging laser by eightfold. As shown in Fig. 3g, the decay of cos(θ) was indeed accelerated in the stronger field for both control and wortmannin-treated cells; however, the defect beyond 30 s remained unchanged. Therefore, beyond promoting cell polarization, PI3K is required for persistent migration but not necessarily for direction sensing *per se*.

### Light-guided migration and relocation of T cells *in vivo*

**Figure 4:**
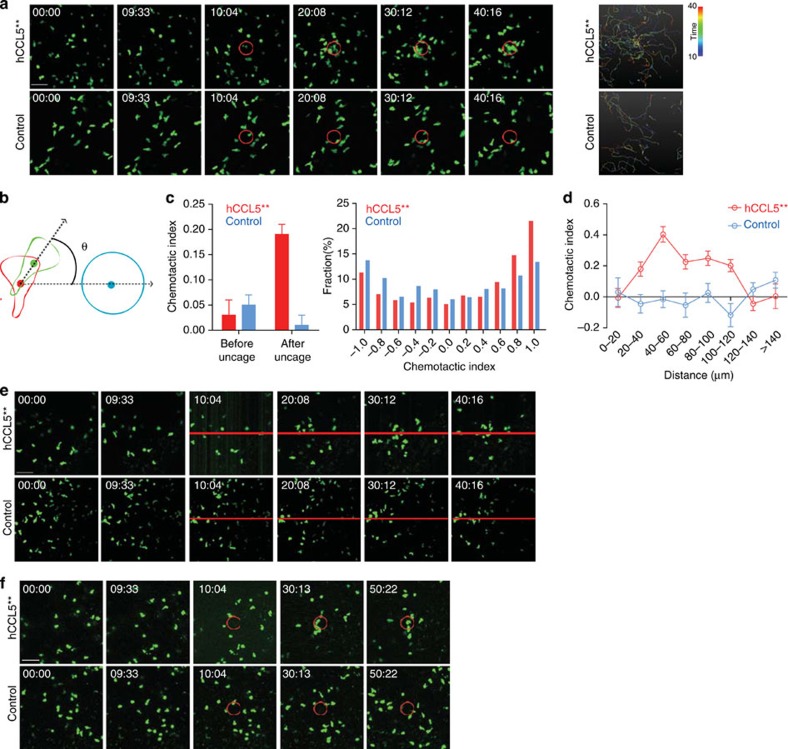
Light-guided migration and relocation of T cells *in vivo*. (**a**–**d**) Uncaging of hCCL5** and directional T-cell migration in dermal tissues. (**a**) Time-lapse images (left) and time-coded tracks (right) of T cells migrating in dermal tissues of the ear injected with hCCL5** or PBS (control) before and after uncaging by raster-scanning with 720-nm light in the marked area (red circle). Cell tracks during the first 20 min immediately after the onset of uncaging. Scale bar, 50 μm. (**b**) Definition of the chemotactic index (CI) as the cosine of the angle *θ* between the velocity vector and the vector connecting the cell to the VOI centre. (**c**) Left, average CI of control and hCCL5** group 10 min before and after uncaging; right, CI distribution of cells starting from outside of the VOI during the first 10 min immediately after the onset of uncaging. (**d**) CIs plotted against distances away from the centre of the VOI as in **a**. (**e**, **f**) Directional T-cell migration in the lymph node after uncaging hCCL5** either by a light path patterned as a line (**e**) or as a circle (**f**) on all optical sections (3-μm step). Scale bar, 50 μm. Error bars indicate s.e.m.

When combined with intravital microscopy, a two-photon caged chemokine system could offer the ability to deliberately localize migratory immune cells to desired location *in vivo*. To test this, we first conducted ear skin imaging following intradermal injection of hCCL5** and green fluorescent protein (GFP)-expressing CD8^+^ T cells activated *in vitro*. Similar cell transfer protocols have been used to probe behaviours of neutrophils in inflamed tissues^25^. Typically within 1 h after transfer, CD8^+^ T cells would settle in and migrate in an apparently random manner in the dermal tissue. We used a dual-laser system in which a pulsed 920-nm laser was used to image the GFP^+^ T cells, while a second pulsed 720-nm laser was used to photo-stimulate a pre-defined cylindrical volume of interest (VOI). As shown in Fig. 4a and Supplementary Movie 10, the CD8^+^ T cells were seen to stream into the VOI soon after the onset of uncaging, while these cells remained randomly migrating when PBS instead of hCCL5** was injected. To quantitatively characterize the directionality of CD8^+^ T cells migrating towards the VOI source of active hCCL5, we defined the chemotactic index (CI) as the cosine of the angle between the vector connecting the cell to the VOI centre and the vector of the cell instantaneous velocity (Fig. 4b). Before uncaging, the transferred T cells migrated with an average CI of 0.03. After uncaging, the average CI rose to 0.19 during the first 10 min, with a significant number of cells exhibiting individual CIs close to 1. In contrast, the control group exhibited an average of 0.01 (Fig. 4c). We also observed a relationship between the strength of chemotactic behaviours and the distance from the chemokine source. As shown in Fig. 4d, within the VOI itself (0–20 μm to the centre), cells exhibited an average CI of 0, consistent with the fact that cells do not show directionality in a uniform field of any chemokine. The CI increased to ∼0.4 within the belt 40–60 μm away from the centre and rapidly decreased farther away. At the end of the 50-min imaging and photo-stimulation, the average cell number within the VOI rose from 3 to 8. These data demonstrate a chemotactic gradient can be artificially established *in vivo* through two-photon uncaging of a caged chemokine to acutely relocalize lymphocytes. Finally, we further attempted to manipulate lymphocyte positioning in the lymph node, a critical organ in which many of the fate decisions during lymphocyte effector development are made in a spatiotemporally defined manner. To this end, we adoptively transferred GFP-expressing CD8^+^ T cells intravenously and injected hCCL5** intravenously during the imaging session. As shown in Fig. 4e and Supplementary Movie 11, when uncaging was conducted as a ‘line', essentially a sheet in the *z* direction of the three-dimensional space, directed migration and accumulation of T cells towards the ‘line' were observed. When uncaging was done within a cylinder VOI that was as deep as 100–150 μm below the capsule, T cells were also seen to collectively migrate towards the VOI, with some cells migrating from tissue regions above the upper bound of or below the lower bound of the imaging stack (Fig. 4f and Supplementary Movie 12). Together, these data demonstrate creation of artificial chemotactic fields and on-demand repositioning of lymphocytes *in vivo*.

## Discussion

In this study, we have created a photo-caged chemokine that allows highly efficient, real-time two-photon uncaging *in vitro* and *in vivo*. Chemokines activatable by two-photon illumination represents a novel strategy to study biochemistry and cell biology of cell migration. A distinct advantage is the high spatiotemporal precision it offers. As demonstrated in our study, on very short (<5 s) and focused (<0.1 μm^2^) 720-nm two-photon irradiation, uncaged hCCL5** induces pseudopod dynamics in CD8^+^ T lymphocytes, in principle enabling studies of chemokine-triggered signalling events inside single pseudopod with a temporal resolution of seconds. Another important advantage of the new system is that it can be used *in vivo* when combined with intravital two-photon microscopy. Indeed, using the two-photon-activatable hCCL5**, we have demonstrated its application in artificial lymphocyte repositioning *in vivo*, both in dermal tissues and deep inside the lymph node. The *in-vitro* findings that PI3K is not required for direction sensing *per se* but for persistent migration of lymphocytes, as revealed here at the single-cell level by the new experimental system, are in good agreement with observations *in vivo* that PI3K activities continuously triggered by non-G-protein-coupled-receptor receptors facilitate chemokine-guided directional migration by promoting persistent motility^23^, and support a more pseudopod-centred view than a strict compass-based model of chemotaxis^3^,^4^,^22^. On the other hand, much faster imaging instrument has to be developed to take full advantage of the spatiotemporal controllability offered by the caged agent to study such single-cell kinetic responses to chemokines in the true three-dimentional tissue space *in vivo*. Our work also suggests potential application of two-photon-activatable chemokines in studies of chemokine signalling during synapse/kinapse formation. Therefore, our caged chemokine system, by allowing artificial cell repositioning, opens new avenues for studying immune cell dynamics and spatiotemporal regulation of cell fate determination *in vivo*.

Our results indicate that caging of more than one amino acid residues is needed to develop a tightly controlled photo-activatable protein agent. Whereas incorporation of multiple tailor-designed amino acids is difficult to accomplish with recombinant engineering technology, direct chemical synthesis^26^ is particularly suited for such tasks. The activity-caging strategy presented in this work is to modify key residues of the CCL5 chemokine ligand–receptor interaction interface. To generalize the strategy to any chemokines or other types of protein ligands, the only information needed is molecular details of the protein–protein interaction interface obtainable by either alanine scan mutagenesis or crystal structure analysis. For small- to medium-sized proteins or protein domains (for example, <200 amino acid), technology demonstrated here should enable synthesis in the multi-milligram scale, sufficient for both *in-vitro* and *in-vivo* studies.

Intercellular communication among different cell types through secreted factors such as chemokines, cytokines and hormones represents one of the most sophisticated and complex aspects of tissue physiology. The strategy of synthetic chemistry combined with a live-imaging approach, as developed and adopted in the present study, demonstrates a novel platform to interrogate responses of single cells to any protein factors in physiological tissue environment in a spatiotemporally defined manner.

## Methods

### Peptide synthesis

All peptides were manually synthesized using standard Fmoc protocols. The 2-Cl-(Trt)-NHNH_2_ resin was prepared directly from 2-Cl-(Trt)-Cl resin by treatment with 5% NH_2_NH_2_ in *N*,*N*-dimethylformamide (DMF) two times, 30 min for each time to prepare the corresponding peptide hydrazides. An enamide motif^27^ attached to Rink Amide resin was used for peptide thioester synthesis. For each coupling reaction, the resin was coupled with amino acid (4 eq.), 2-(6-chloro-1*H*-benzotriazole-1-yl)-1,1,3,3-tetramethylaminiumhexafluorophosphate (HCTU)/1-[bis(dimethylamino)methylene]-1*H*-1,2,3-triazolo-[4,5-b]pyridinium hexafluorophosphate 3-oxide (HATU) (3.6 eq.) in 0.25 M *N*,*N*-diisopropylethylamine (DIEA) solution. Proper coupling conditions were chosen for each amino acid, including the number of coupling reactions (for example, single or double coupling) and the type of coupling reagent (HCTU/HATU). A single-coupling reaction using HCTU for 60 min was enough for most of the amino acids. For sterically hindered amino acids (such as Fmoc-Asn(Trt)-OH, Fmoc-Ile-OH, Fmoc-Gln(Trt)-OH, Fmoc-Arg(Pbf)-OH, Fmoc-Val-OH and Fmoc-Thr(tBu)-OH) and the amino acid right after Pro, a double-coupling strategy was recommended. For Fmoc-Ser(DMNB)-OH and Fmoc-Ser(NDBF)-OH, the photo-sensitive amino acid (1.5 eq.), HATU (1.5 eq.) and HOAt (1.5 eq.) in 0.5 M DIEA/DMF solution was used for coupling. Twenty per cent piperidine in DMF (v/v) was used to remove the Fmoc protecting group. The overall deprotection of side-chain protecting groups and final cleavage from the resin were achieved by trifluoroacetic acid cocktails (88% trifluoroacetic acid, 5% phenol, 2% triisopropylsilane, 5% H_2_O) for about 2 h at room temperature (r.t.). The combined solutions were concentrated by blowing with N_2_ and then treated with cold dry ether. The crude peptides obtained after centrifugation were dissolved in CH_3_CN/H_2_O for further analysis and purification. Peptides were confirmed by matrix assisted laser desorption/ionization–time-of-flight–mass spectrometry (MS) or electrospray ionization–MS.

### A one-pot synthesis of hCCL5 and its caged analogues.

*(A) Native chemical ligation of [Thz^11^-Lys^33^]^α^thioester and [Cys^34^-Ser^68^] and Thz-to-Cys conversion:* [Thz^11^-Lys^33^]^α^thioester (1.0 eq.) and [Cys^34^-Ser^68^] (1.0 eq.) were dissolved in ligation buffer (6 M Gn·HCl, 200 mM Na_2_HPO_4_, 100 mM 4-mercaptophenylacetic acid (MPAA), 20 mM tris(2-carboxyethyl)phosphine hydrochloride (TCEP), pH 6.8), to reach a final concentration of about 4 mM. The resulting solution was stirred at r.t. for 4 h. Solid MeONH_2_·HCl (with the final concentration of 0.25 M) was added and the pH was adjusted using 6 M HCl to about 4.0. The reaction was continued for another 4 h to convert Thz-peptide to Cys-peptide.

*(B) Oxidation and thioesterification of [Ser^1^-Cys^10^]-NHNH_2_ and its caged forms:* Each peptide hydrazide (1.2 eq.) was dissolved at a concentration of 4 mM in 0.2 M phosphate solution containing 6 M Gn·HCl, pH 3.0. The solution was placed in a −15 °C ice-salt bath and gently agitated by magnetic stirring. Ten eq. NaNO_2_(0.5 M) to the peptide was added to oxidize the peptide hydrazide to azide within 20 min. Subsequently, 200 mM MPAA (dissolved in 0.2 M phosphate solution containing 6 M Gn·HCl, pH 6.0) with equal volume was pipetted to the reaction to eliminate excess NaNO_2_ and convert the peptide azide to the corresponding peptide thioester. The reaction was then removed from the ice-salt bath and kept at r.t. for 10 min. It is noteworthy that on the condition of [Ser^1^-Cys^10^(Ser_1,5_DMNB)]-MPAA and [Ser^1^-Cys^10^(Ser_1,5_NDBF)]-MPAA, 20% (v/v), 2,2,2-trifluoroethanol (TFE) was added to solve the solubility problem. The thioester of Segment 1 can also be isolated and lyophilized for the second ligation.

*(C) Native chemical ligation of [Ser^1^-Cys^10^]-MPAA (or its caged forms) and [Cys^11^-Ser^68^]:* Solution containing [Ser^1^-Cys^10^]-MPAA (or its caged forms) was entirely added to the reaction mixture containing [Cys^11^-Ser^68^] and the pH was adjusted to 6.8. This mixture was continued to react for another 2 h, to generate to full-length hCCL5 (or its caged forms). The whole process was monitored by liquid chromatography–MS. The final product was purified by semi-preparative HPLC and lyophilized.

### Refolding of hCCL5 and its caged analogues

Purified hCCL5 and its caged analogues were dissolved in an aqueous buffer of 2 M Gn·HCl and 100 mM Tris containing 8 mM cysteine and 1 mM cystine, pH 8.0. The solutions were gently stirred overnight at r.t., purified by semi-preparative HPLC and confirmed by electrospray ionization–MS.

### Ultraviolet photolysis of caged peptides

Photolysis by ultraviolet irradiation was performed on Omnicure S1500 (EXFO Photonic Solutions Inc., Canada) with a 365-nm filter, at a light intensity of 18 mW cm^−2^. Purified peptides with photo-sensitive groups were dissolved in phosphate buffer containing 6.0 M Gn·HCl, pH 7.4. Analytical HPLC was used to analyse the results of irradiation with varying lengths of time: 0, 5, 10, 30, 60, 120, 180, 240 and 300 s. Benzyl amide as the internal reference standard was used for quantification of reactant and product.

### Mice

B6 (Jax 664), albino B6 (Jax 058), GFP-expressing B6 (Jax 4353), dsRed-expressing B6 (Jax 6051) and *CCR5*^−/−^ (Jax 5427) mice were from the Jackson Laboratory. OVA_257–264_-specific T-cell receptor transgenic OT-I (Jax003831) was a gift from Dr Yan Shi in Tsinghua University. All mice were maintained under specific pathogen-free conditions and all animal protocols were approved by the Tsinghua Animal Care and Usage Committee in accordance of governmental and institutional guidelines for animal welfare.

### Cell culture

Naive OT-I CD8^+^or polyclonal CD8^+^ cells were isolated using the positive CD8^+^ T-cell isolation kit (Miltenyi Biotec) and polyclonal CD4^+^ cells were isolated using the positive CD4^+^ T-cell isolation kit (Miltenyi Biotec), according to the manufacturer's protocols. For *in-vitro* T-cell activation, T cells were stimulated by 10 μg ml^−1^ plate-bound anti-CD3 and 10 μg ml^−1^ anti-CD28. Following activation for 2 days, T cells were transferred into 75-cm culture flask and maintained with fresh media supplemented with 10 ng ml^−1^ IL-2 (Peprotech) as needed. For imaging and transwell migration experiments, T cells were typically cultured *in vitro* for 4–6 days before use.

### Transwell migration assay

*In vitro*-activated polyclonal CD8^+^ T cells were rested in the RPMI medium containing 1% fetal bovine serum at 37 °C for 1 h before being loaded as a 100-μl suspension of 10^5^ cells into the upper well (5-mm pore, Corning). hCCL5** before or after ultraviolet deprotection, achieved by ultraviolet irradiation (18 mW cm^−2^) for 30 s, was added to the bottom wells at indicated concentrations. Cells that transmigrated into the bottom well were enumerated 3 h later by flow cytometry with an internal cell counting standard.

### Imaging of T-cell migration *in vitro*

DsRed-expressing CD8^+^ T cells were activated *in vitro* and rested in the RPMI medium containing 1% fetal bovine serum at 37 °C on a 35-cm dish (NEST) pre-coated with 7.5 μg ml^−1^ recombinant mouse ICAM-2 (R&D) in PBS for 1 h before imaging. To inhibit PI3K activities, the T cells were pre-treated with 100 nM Wortmannin (Sigma) for 1 h before imaging and maintained in Wortmannin-containing media during imaging. Images were acquired using a Nikon A1RMP microscope equipped with Mai Tai DeepSee laser (Spectra-Physics), a 561-nm laser (Coherent) and × 60 oil objective lens (Nicon). Acquisition was controlled by NIS-Elements software (Nikon). Data were processed by NIS-Elements Analysis software (Nikon) and Imaris (Bitplane).

### Imaging CD8 T-cell interactions with target cells

For cell–cell interaction imaging, *in vitro*-activated CD4^+^ cells were incubated with 20 pg ml^−1^ OVA_257-264_ for 30 min, then washed with 1 × PBS for five times. A total of 10^5^ CD8^+^ T cells and 10^5^ peptide-pulsed CD4^+^ cells were loaded onto the ICAM pre-coated 35-cm dish and rested for 30 min before imaging.

### Two-photon intravital imaging of T-cell chemotaxis

For *in vivo* imaging of T-cell migration in the dermal tissue, ∼10^6^ activated GFP-expressing CD8^+^ T cells were loaded into 20 μl PBS containing 50 ng μl^−1^ hCCL5** and intradermally injected into the ear of albino B6 mice. Imagining was conducted at least 1 h after the injection and the field of view was chosen not to be directly at the injection site. For *in vivo* imaging of T-cell migration in the lymph node, the basic procedure was essentially as described previously^23^. Briefly, 3 × 10^6^ activated GFP-expressing CD8^+^ T cells were intravenously transferred into B6 mice the day before and 20 μg hCCL5** was intraveneously injected 30 min before imaging. An infrared laser tuned to 920 nm (Mai Tai DeepSee, Spectra-Physics) was used to image GFP-expressing cells and a second laser tuned to 720 nm to photostimulate the region of interest. Photostimulation was always in a continuous mode in sync with imaging. Four-dimensional data sets were analysed using Imaris software (Bitplane). Cell migration was analysed by Imaris cell tracking module aided with manual correction and verification. Cell tracks that lasted for <1 min were excluded from analysis. For analysis of the mean chemotactic index within 10 min of photostimulation, cells with a path length of <50 μm were excluded, because they often leave the imaging volume too soon. AfterEffects and Illustrator were used to produce time-lapse image sequences and movies.

### Statistical analysis

Unless indicated otherwise, *t*-tests were used to compare endpoint means of different groups. Statistic test and graphing were done with Prism (GraphPad).

## Additional Information

**How to cite this article**: Chen, X. *et al.* Chemical synthesis of a two-photon-activatable chemokine and photon-guided lymphocyte migration *in vivo*. *Nat. Commun.* 6:7220 doi: 10.1038/ncomms8220 (2015).

## Supplementary Material

Supplementary Figures, Methods and References Supplementary Figures 1-8, Supplementary Methods and Supplementary References

Supplementary Movie 1 A dsRed-expressing CD8+ T cell chasing the focus of a 720-nm 2- photon excitation laser in media containing hCCL5** Red dots show the centroid positions of the cell and green dots show previous positions of the laser focus. Green cross follows current laser focus where uncaging took place. Also see Figure 2a.

Supplementary Movie 2 CD8+ T cell accumulation toward the uncaging light path patterned in a heart shape in hCCL5**-containing media. The red curve indicates the uncaging light path. Uncaging was by a 720 nm laser in sync with imaging by a 561 nm laser. Also see corresponding time-lapse images in Figure 2b.

Supplementary Movie 3 A stable contact engaged CD8+ T cell response to hCCL5 attraction delivered by a focus of 720-nm 2-photon excitation. Red dots: previous positions of the laser focus; Red cross: current focus at the indicated time point. Scale bar, 10μm.Also see corresponding time-lapse images in Figure 2c.

Supplementary Movie 4 An unstable contact engaged CD8+ T cell response to hCCL5 attraction delivered by a focus of 720-nm 2-photon excitation. Red dots: previous positions of the laser focus; Redcross: current focus at the indicated time point. Scale bar, 10μm. Also see corresponding time-lapse images in Figure 2d.

Supplementary Movie 5 Chemotaxis of untreated CD8+ T cells toward the uncaging light path patterned as a line in hCCL5**-containing media. The uncaging was started at the second minute and was synchronized with image acquisition thereafter. Also see corresponding time-lapse images in Figure 3a.

Supplementary Movie 6 Chemotaxis of untreated CD8+ T cells toward the uncaging light path patterned as a line in hCCL5**-containing media. The uncaging was started at the second minute and was synchronized with image acquisition thereafter. Also see corresponding time-lapse images in Figure 3a.

Supplementary Movie 7 A CD8+ T cell that generated a new pseudopod in response to active CCL5 in hCCL5**-containing media (N-turn). Red cross follows current laser focus where uncaging took place. Also see corresponding time-lapse images in Figure 3c.

Supplementary Movie 8 A CD8+ T cell that extended its existing pseudopod toward active CCL5 in hCCL5**-containing media (U-turn). Red cross follows current laser focus where uncaging took place. Also see corresponding time-lapse images in Figure 3d.

Supplementary Movie 9 A CD8+ T cell that was uncommitted for a prolonged period in response to active CCL5 (C-turn). Red cross follows current laser focus where uncaging took place. Also see corresponding time-lapse images in Figure 3e.

Supplementary Movie 10 Directional T cell migration in response to hCCL5** uncaging in dermal tissues. GFP-expressing CD8+ T cells migration in the ear dermis visualized before and after uncaging by a 720-nm laser scanning the area marked by the red circle. Also see corresponding time-lapse images in Figure 4a. Scale bar, 50 μm.

Supplementary Movie 11 Directional CD8+ T cell migration in response to hCCL5** uncaging in the lymph node by a light path patterned as a line. GFP-expressing CD8+ T cells migration in the lymph node visualized before and after uncaging by a 720-nm laser scanning the red line. Also see corresponding time-lapse images in Figure 4e. Scale bar, 50 μm.

Supplementary Movie 12 Directional CD8+ T cell migration in response to hCCL5** in the lymph node uncaged in a circular area. GFP-expressing CD8+ T cells migration in the lymph node visualized before and after uncaging by a 720-nm laser scanning the area within the red circle. Also see corresponding time-lapse images in Figure 4f. Scale bar, 50 μm.
