# Supplementary material for: Chemical synthesis of a two-photon-activatable chemokine and photon-guided lymphocyte migration in vivo
Source: Nat Commun. 2015 May 26;6:7220. doi: 10.1038/ncomms8220 (PMC4455097; doi:10.1038/ncomms8220)
Supplement: Supplementary Figures, Methods and References — Supplementary Figures 1-8, Supplementary Methods and Supplementary References [file ncomms8220-s1.pdf]

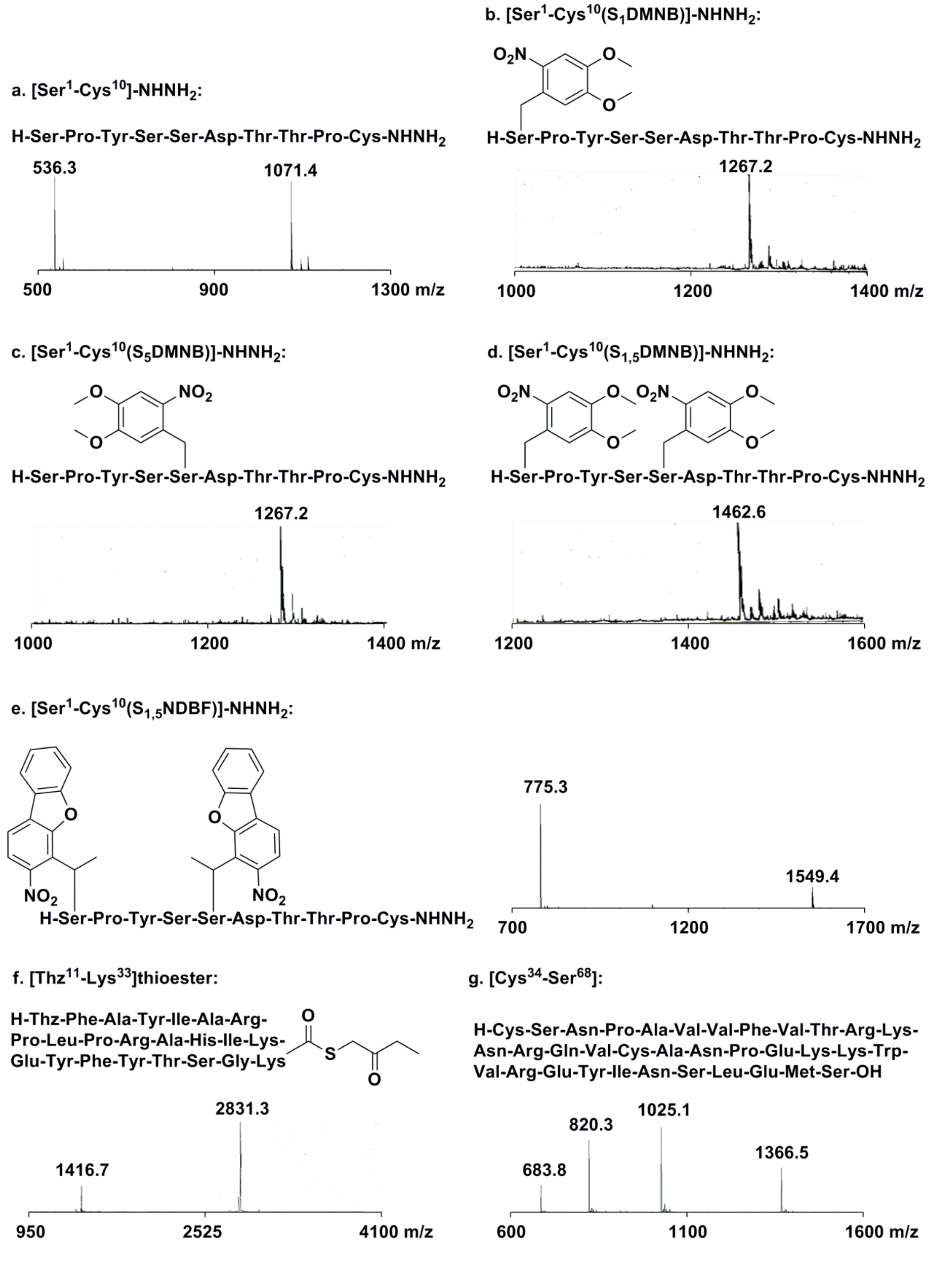

Supplementary Figure 1. Structural formula and mass data of segments of hCCL5. Structural formula and the mass of (a) [Ser^{1}-Cys^{10}]-$NHNH_2$, (b) [Ser^{1}-Cys^{10}(S_1DMNB)]-$NHNH_2$, (c) [Ser^{1}-Cys^{10}(S_5DMNB)]-$NHNH_2$, (d) [Ser^{1}-Cys^{10}($S_{1,5}$DMNB)]-$NHNH_2$, (e) [Ser^{1}-Cys^{10}($S_{1,5}$NDBF)]-$NHNH_2$, (f) [Thz^{11}-Lys^{33}]$^{\alpha}$thioester, (g) [Cys^{34}-Ser^{68}].

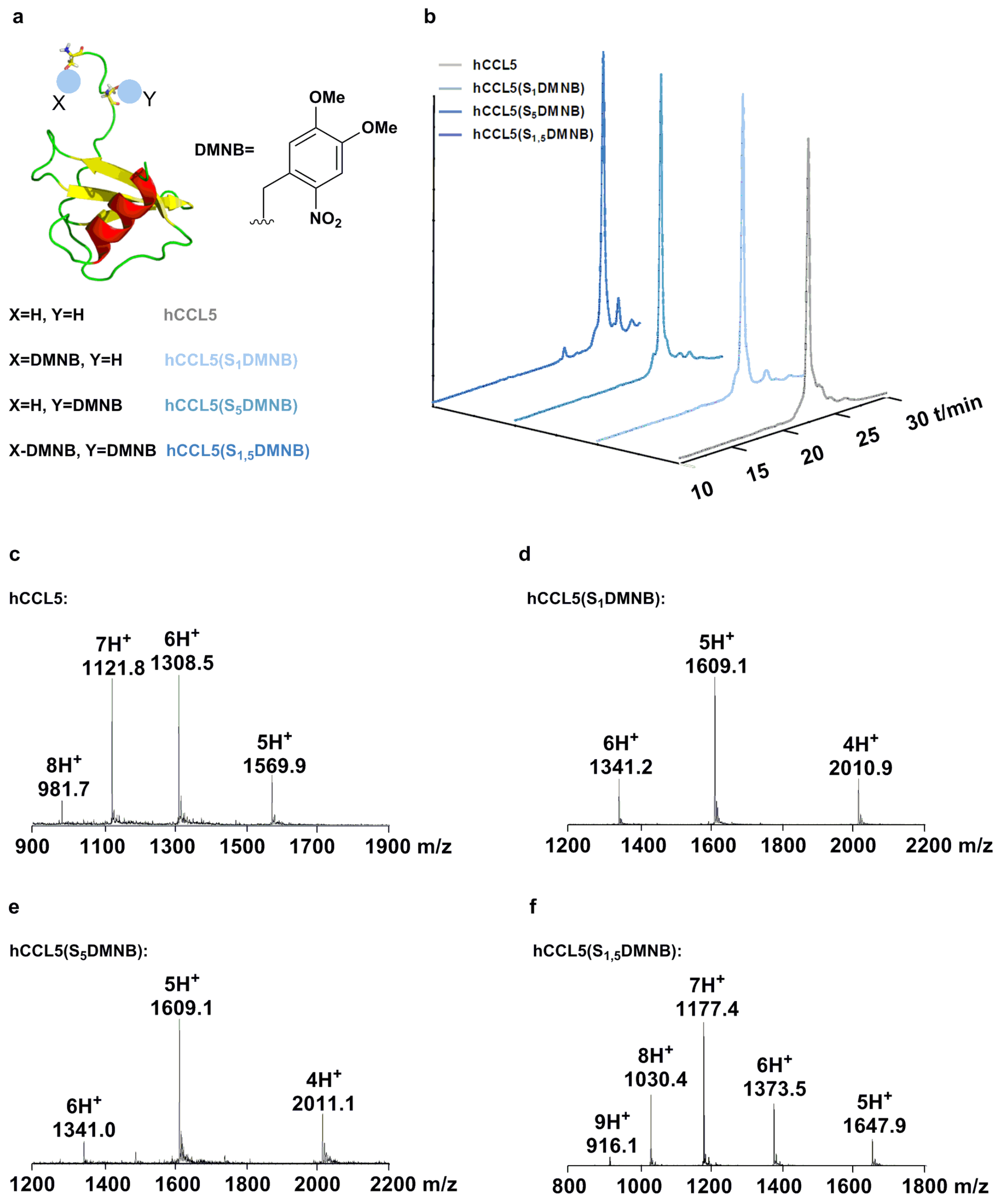

Supplementary Figure 2. Structure, synthesis and characterization of hCCL5 and DMNB caged versions. (a) Structures of hCCL5 and DMNB caged forms. All of the versions were synthesized according to the same strategy described in Figure 1a. (b) HPLC traces of the refolding step of hCCL5 and DMNB caged forms. (c) ESI-MS of folded hCCL5, singly caged hCCL5(S_1DMNB),hCCL5(S_5DMNB) and doubly caged hCCL5($S_{1,5}$DMNB).

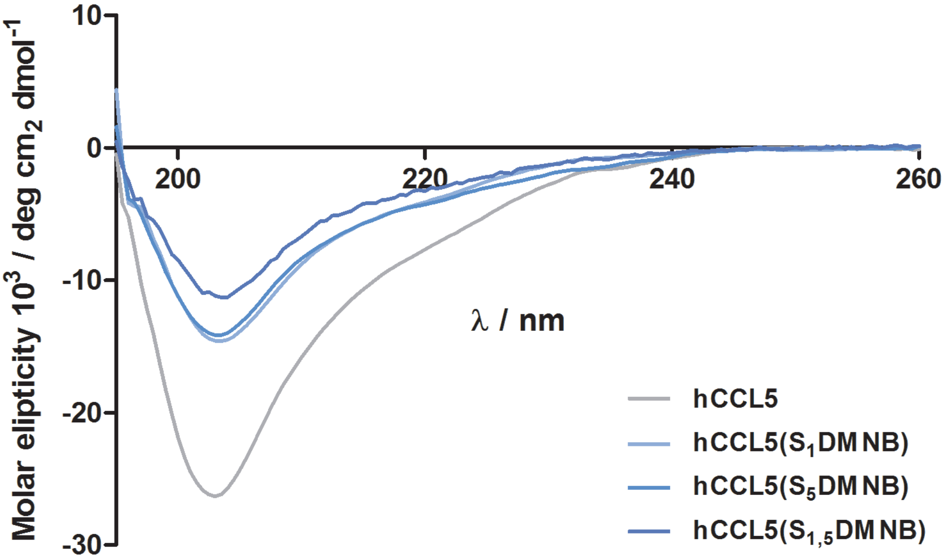

Supplementary Figure 3. CD spectra of wild type hCCL5, singly caged hCCL5(S_1DMNB), hCCL5(S_5DMNB) and doubly caged hCCL5($S_{1,5}$DMNB). Data points in a wavelength range of 195-260 nm were collected. The spectrum for each version was performed in triplicate, averaged, subtracted from blank and smoothed.

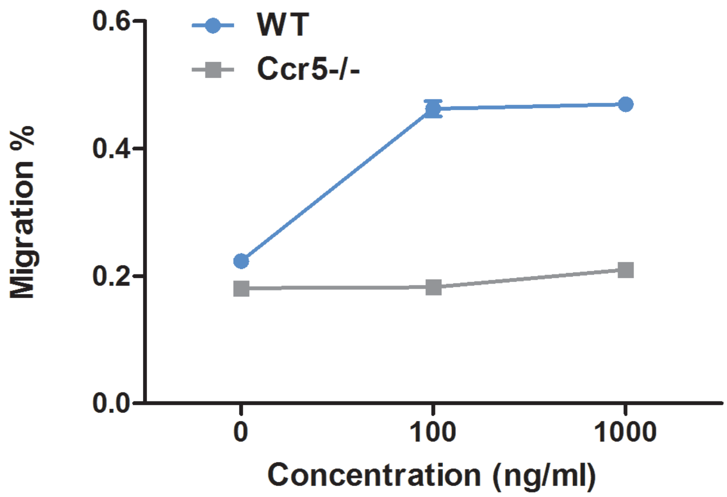

Supplementary Figure 4. Chemotaxis assay for murine *Ccr5*$^{+/+}$ or *Ccr5*$^{-/-}$ CD8^{+} T lymphocytes. Synthetic WT hCCL5 of 100 ng/ml and 1000 ng/ml were used to perform the transwell migration assay. The mean of three experiments is plotted, and the standard deviation is represented by error bars.

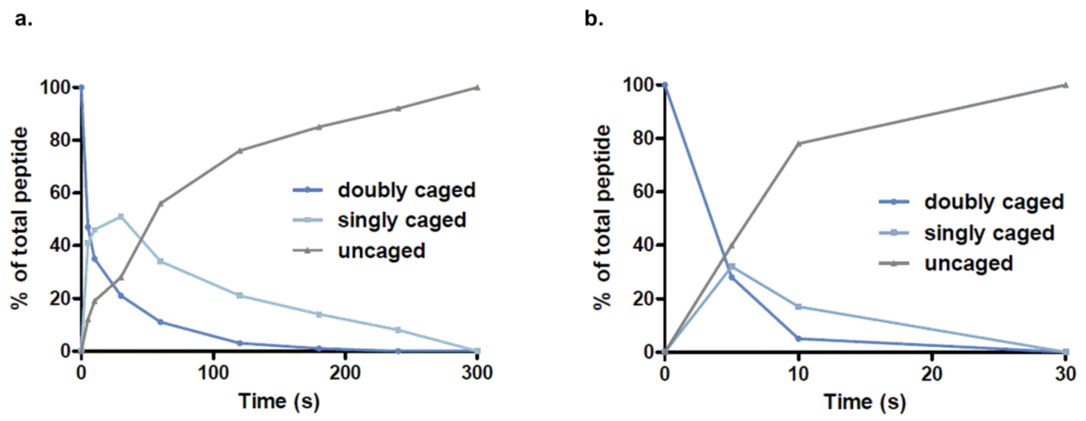

Supplementary Figure 5. Photolysis kinetics of doubly caged peptides. (a) Photolysis of [Ser[1]-Cys[10](Ser$_{1,5}$DMNB)]-$NHNH_2$. (b) Photolysis of [Ser[1]-Cys[10](Ser$_{1,5}$NDBF)]-$NHNH_2$. Analytic HPLC was used to quantify the doubly caged (●), singly caged (■), and uncaged forms (▲).

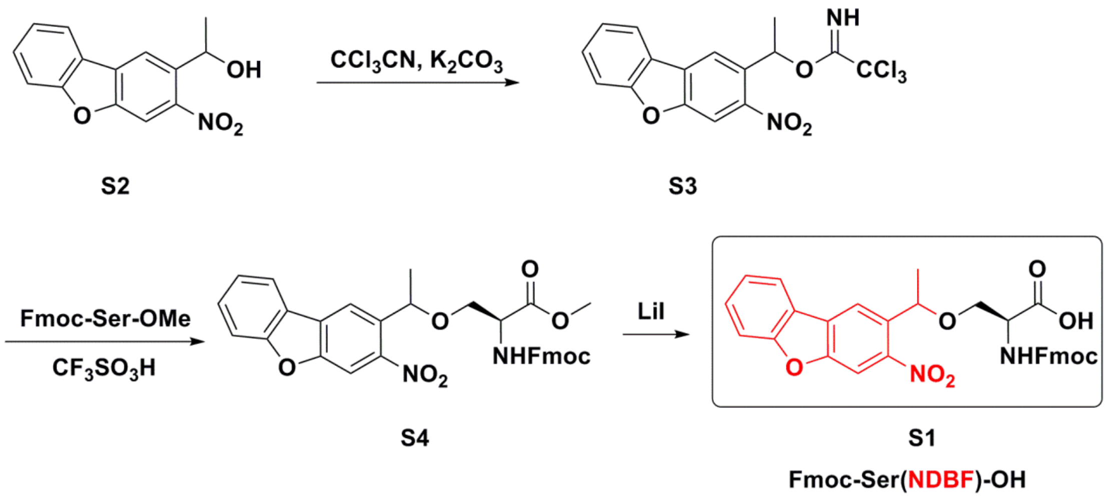

Supplementary Figure 6. Synthetic route for Fmoc-Ser(NDBF)-OH starting from 1-(3-Nitrodibenzofuran-2-yl)-ethanol.

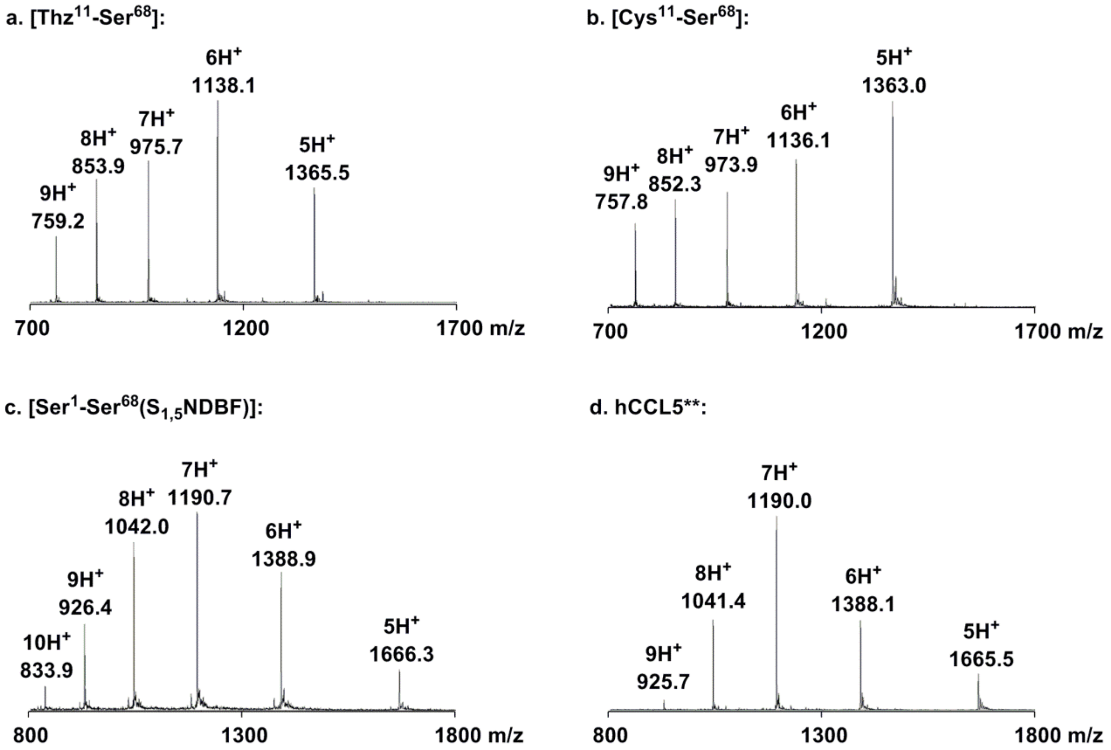

Supplementary Figure 7. The mass data of intermediates and final products during one-pot synthesis of hCCL5**. ESI/MS of (a) intermediates[Thz^{11}-Ser^{68}], (b) [Cys^{11}-Ser^{68}], (c) [Ser^{1}-Ser^{68}($S_{1,5}$NDBF)], and (d) the final folded two-photon caged product hCCL5**.

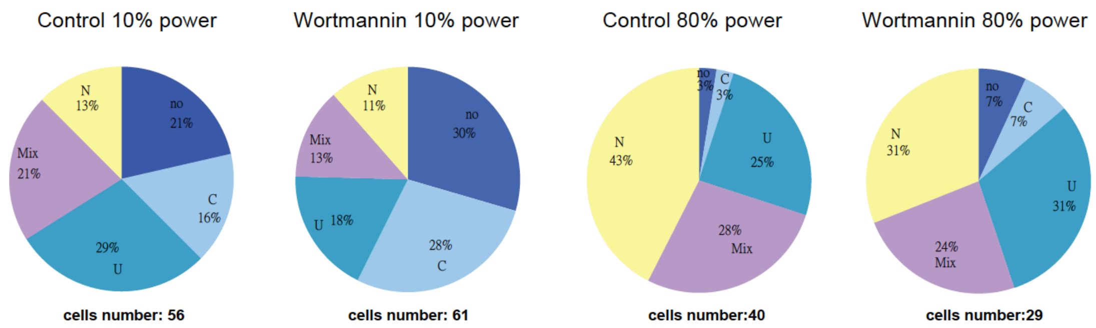

Supplementary Figure 8. Frequencies of different turn behaviors of T cells in acute response to a point source of active hCCL5 *in vitro*. T-cell behaviors classified according to examples in Figs. 3c-e as N-turn (N), U-turn (U), C-turn (C), N- and U-turn hybrid (Mix), or no response (no), following stimulation by a point source of active hCCL5 in the presence or absence of 100 nM wortmannin. See corresponding Fig. 3 and Supplementary Movie 7-9 for additional details.

Supplementary Methods

Synthesis of Fmoc-Ser(NDBF)-OH.

We developed an optimized synthetic route to Fmoc-Ser(NDBF)-OH exhibited in Supplementary Figure 6. With 1-(3-Nitrodibenzofuran-2-yl)-ethanol (**S2**, synthesis according to a previous report[1]) in hand, we obtained the target molecule in three steps[2,3]. The details were as follows:

S2 (514 mg, 2 mmol) was dissolved in 10 mL of anhydrous methylene chloride and stirred at room temperature under Ar. Anhydrous K_2CO_3 (830 mg, 6 mmol), trichloroacetonitrile (840 mg 4 mmol), and triethylamine (300 mg, 3mmol) were added and the reaction mixture was kept to stir for 24 h. 20 mL methylene chloride was added to the mixture, and the suspension sequentially washed with 1 M HCl and saturated NaCl. The organic layer was dried (Na_2SO_4) and concentrated under reduced pressure. The residue was purified by flash chromatography with petroleum ether / ethyl acetate (PE/EtOAc=30:1) to afford the desired compound as a dark brown powder (360 mg, 45%): ^{1}H NMR (400 MHz, $CDCl_3$): δ 8.30 (s, 1H), 8.27 (s, 1H), 8.20 (s, 1H), 7.96 (d, J = 7.8 Hz, 1H), 7.63 (d, J = 8.0 Hz, 1H), 7.58 (t, J = 7.8 Hz, 1H), 7.43 (d, J = 8.0 Hz, 1H), 6.56 (q, J = 6.4 Hz, 1H), 1.86 (d, J = 6.5 Hz, 3H); ^{13}C NMR (400 MHz, $CDCl_3$) δ 160.94, 158.52, 152.02, 146.38, 133.21, 129.71, 129.49, 123.97, 122.66, 121.77, 118.68, 112.45, 108.56, 91.95, 73.29, 22.44.

Triflic acid (15 μL) was added to an anhydrous methylene chloride (10 mL) solution of Fmoc-Ser-OMe (460 mg, 1.35 mmol) and acetimidate **S3** (360 mg, 0.9 mmol) kept under Ar at room temperature. The resulting dark brown solution was stirred for 20 min. Further addition of triflic acid (15μL) was carried out twice at 20 min intervals. The mixture was continued to react for another 2 h. 10 mL methylene chloride was added to the reaction mixture, and the suspension was washed with saturated NaCl twice, dried, and concentrated under reduced pressure. The crude residue was purified by flash chromatography with petroleum ether / ethyl acetate (PE/EtOAc=8:1) to afford product **S4** as a brown powder (diastereoisomers, 160 mg, 30%). Light should be taken care to avoid photo-release of the NDBF group. ^{1}H NMR (300 MHz, $CDCl_3$) δ 7.76-7.01 (m, 15H), 5.73-5.60 (m, 1H), 4.99-4.92 (m, 1H), 4.50-4.15 (m, 4H), 3.86-3.56(m, 4H), 1.62-1.46 (m, 3H). ^{13}C NMR (300 MHz, $CDCl_3$) δ 170.75, 158.45, 157.57, 156.00, 155.39, 149.06, 143.97, 143.82, 141.41, 132.76, 130.35, 128.95, 127.84, 127.18, 125.22, 125.06, 123.13, 123.05, 120.11, 119.98, 113.18, 113.11, 108.58, 73.81, 69.08, 67.31, 54.74, 52.74, 47.20, 23.34.

To a dry ethyl acetate solution (5 mL) of purified **S4** (160mg, 0.275 mmol), Lithium iodide (295 mg, 2.2 mmol) was added under Ar. The mixture was under reflux for about 12 h. The reaction was quenched by the addition of 10 mL 1 M HCl. The resulting solution was extracted with EtOAc. The organic extract was sequentially washed with 5% aqueous $Na_2S_2O_3$ solution, 1M HCl, saturated NaCl, and dried (Na_2SO_4), filtered and evaporated in vacuum. The brown solid was purified by column chromatography (CH_2Cl_2/MeOH, 30:1) to afford the desired product **S1** (diastereoisomers, 140 mg, 90%). ^{1}H NMR (400 MHz, $CDCl_3$) δ 8.14-7.13 (m, 15H), 5.79-5.63 (m, 1H), 5.18-4.96 (m, 1H), 4.56-4.17 (m, 4H), 3.81-3.66(m, 1H), 1.68-1.49 (m, 3H). ^{13}C NMR (400 MHz, $CDCl_3$) δ 172.96, 159.96, 158.26, 156.1, 153.90, 146.71, 143.64, 143.28, 141.33, 133.95, 129.45, 127.84, 127.15, 125.06, 123.61, 122.34, 121.97, 120.05, 120.02, 118.94, 112.09, 108.43, 74.41, 68.91, 68.71, 54.36, 46.95, 21.04. The mass of Fmoc-Ser(NDBF)-OH, calcd: $C_{32}H_{26}N_2O_8$ $[M+H]^+$ m/z = 566.6 Da, found: 567.0 Da.

Supplementary Reference

1. Lusic, H.; Uprety, R.; Deiters, A. Improved synthesis of the two-photon caging group 3-nitro-2-ethyldibenzofuran and its application to a caged thymidine phosphoramidite. *Org. Lett.* **12**, 916-919 (2010).
2. Zheng, J.-S.; Yu, M.; Qi, Y.-K.; Tang, S.; Shen, F.; Wang, Z.-P.; Xiao, L.; Zhang, L.; Tian, C.-L.; Liu, L. Expedient total synthesis of small to medium-sized membrane proteins via Fmoc chemistry. *J. Am. Chem. Soc.* **136**, 3695-3704 (2014).
3. Veldhuyzen, W. F.; Nguyen, Q.; McMaster, G.; Lawrence, D. S. A light-activated probe of intracellular protein kinase activity. *J. Am. Chem. Soc.* **125**, 13358-13359 (2003).
